# SARS-CoV-2 seroprevalence at urban and rural sites in Kaduna State, Nigeria, during October/November 2021, immediately prior to detection of the Omicron variant

**DOI:** 10.1093/ije/dyac141

**Published:** 2022-06-28

**Authors:** Gloria D Chechet, Jacob K P Kwaga, Joseph Yahaya, Harry Noyes, Annette MacLeod, Walt E Adamson

**Affiliations:** Department of Biochemistry, Faculty of Life Sciences, Ahmadu Bello University, Zaria, Nigeria; Africa Centre of Excellence for Neglected Tropical Diseases and Forensic Biotechnology, Ahmadu Bello University, Zaria, Nigeria; Africa Centre of Excellence for Neglected Tropical Diseases and Forensic Biotechnology, Ahmadu Bello University, Zaria, Nigeria; Department of Veterinary Public Health and Preventive Medicine, Faculty of Veterinary Medicine, Ahmadu Bello University, Zaria, Nigeria; Department of Biochemistry, Faculty of Life Sciences, Ahmadu Bello University, Zaria, Nigeria; Africa Centre of Excellence for Neglected Tropical Diseases and Forensic Biotechnology, Ahmadu Bello University, Zaria, Nigeria; Centre for Genomic Research, University of Liverpool, Liverpool, UK; Institute of Biodiversity, Life Sciences, and Animal Health, University of Glasgow, Glasgow, UK; Institute of Biodiversity, Life Sciences, and Animal Health, University of Glasgow, Glasgow, UK

**Keywords:** SARS-CoV-2, Covid-19, seroprevalence, Kaduna, Nigeria

## Abstract

**Background:**

Nigeria is Africa’s most populated country. By November 2021 it had experienced three waves of SARS-CoV-2 infection. Peer-reviewed seroprevalence data assessing the proportion of the Nigerian population that have been infected were extremely limited.

**Methods:**

We conducted a serosurvey in one urban site (*n *=* *400) and one rural site (*n *=* *402) in Kaduna State, Nigeria between 11 October 2021 and 8 November 2021. Z-tests were used to compare seroprevalence across age groups, locations and sexes. T tests were used to determine whether age or household size are associated with seropositivity. Associations between seropositivity and recent history of common Covid-19 symptoms were tested using logistic regression.

**Results:**

SARS-CoV-2 antibodies were detected in 42.5% an 53.5% of participants at the urban and rural sites, respectively The overall age- and sex- stratified seroprevalence was 43.7% (42.2% for unvaccinated individuals). The data indicate an infection rate in Kaduna State ≥359-fold the rate derived from polymerase chain reaction-confirmed cases. In the urban site, seroprevalence among females and participants aged <20 was lower than other groups. Reporting loss of sense of taste and/or smell was strongly associated with seropositive status. Associations with seropositivity were also found for the reporting of dry cough, fever, headache, nausea and sore throat.

**Conclusions:**

This study provides baseline SARS-CoV-2 seroprevalence in Kaduna State, Nigeria, immediately prior to the spread of the Omicron variant. It indicates that in October/November 2021, approximately 56% of the population did not have detectable antibodies, and population subgroups with particularly low seroprevalence remain. It highlights limitations in using PCR-confirmed cases to estimate infection rates. The data will inform public health strategies in Nigeria and other sub-Saharan African countries with limited SARS-CoV-2 seroprevalence data.

Key Messages

- We present updated SARS-CoV-2 seroprevalence data for Nigeria, Africa’s most populated country: the first such peer-reviewed data since December 2020.
- By November 2021, SARS-COV-2-reactive antibodies were detected in approximately half of the population surveyed.
- We estimate that in Kaduna State, Nigeria, at least 3.48 million people had been exposed to the virus by the time of surveillance: 359 times more than estimates based on PCR-confirmed cases.
- Our data were collected immediately prior to the spread of the Omicron variant, therefore they provide a baseline from which the burden of the Omicron variant can be estimated.

## Introduction

COVID-19 (caused by SARS-CoV-2 infection) was declared a pandemic by the World Health Organization (WHO) on 11 March 2020.^1^ By November 2021, 256 million people across the world had confirmed infection, and 5.1 million people had died as a result.^2^

Serological antibody tests to detect past exposure to SARS-CoV-2 are a valuable tool for assessing the proportion of a population that have been infected with the virus. They can detect evidence of infection from 2 weeks to several months following infection in both symptomatic and asymptomatic individuals. Identifying SARS-CoV-2 seroprevalence in a population has important implications for public health policy: in determining the effectiveness of measures put in place to prevent the spread of the virus, providing evidence on whether further significant spread remains possible and informing on deployment strategies for testing, treatment and vaccination. Worldwide as of 17 February 2022, 1923 SARS-CoV-2 serological surveys had been published in peer-reviewed journals.^3^ However, serological data for sub-Saharan Africa remain limited and often restricted to subgroups that might not represent the overall population such as health care workers. Since the beginning of the pandemic, several peer-reviewed SARS-CoV-2 seroprevalence studies have been conducted in sub-Saharan Africa.^3^ However, many of the data focus on earlier stages of the pandemic and recent data are limited. There have been just six peer-reviewed surveys published that describe seroprevalence data collected in sub-Saharan Africa in the 6 months prior to the work described here, five which were in Eastern and Southern Africa.^4–9^ Usuf and Roca called for SARS-CoV-2 seroprevalence studies to be carried out in sub-Saharan Africa ‘whenever possible’.^10^

**Figure 1:**
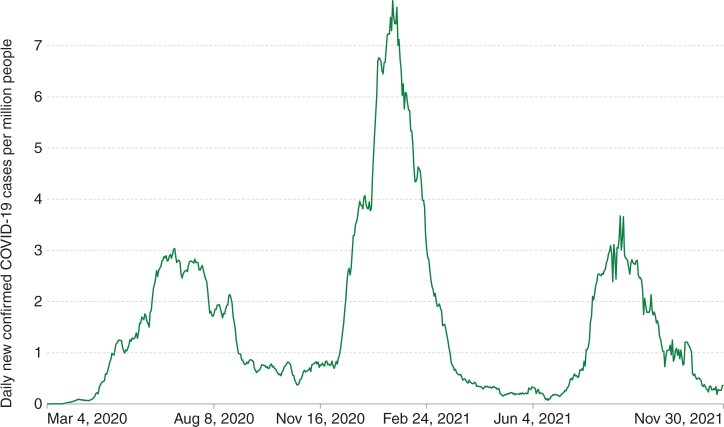
Daily new confirmed COVID-19 cases per million people in Nigeria, 7-day rolling average, 4 March 2020 to 30 November 2021. (Source: *Our World In Data*^2^*.*)

Nigeria, in West Africa, is the continent’s most populated country with approximately 213 million inhabitants. As of November 2021, it had experienced three waves of SARS-CoV-2 infection with peak infection rates occurring in July 2020, January 2021 and August 2021^2^ (Figure 1). By 22 November 2021, official figures reported 213 589 confirmed cases and 2974 deaths in Nigeria. However the per-capita testing rate was substantially lower than the African average (0.02 tests/capita in Nigeria compared with 0.06/capita continent-wide).^2^ In Nigeria there has been one peer-reviewed SARS-CoV-2 seroprevalence survey, examining samples collected in Anambra State between 8 and 15 December 2020 (prior to the second wave of infection) and reporting an overall seroprevalence of 16.1%.^11^ Between 15 December 2020 and 22 November 2021 there were a further 139 457 confirmed SARS-CoV-2 cases in Nigeria (65.3% of the country’s total confirmed cases at that time).^2^ The country’s vaccination programme commenced in March 2021, and by 9 December 2021, 6.08 million inhabitants (3.48% of the population) had received at least one dose of a vaccine.^2^ Earlier surveys in sub-Saharan Africa have highlighted contrasts in SARS-CoV-2 seroprevalence between urban and rural settings, with higher seroprevalence recorded in urban settings in the initial stages of the pandemic, and populations in rural settings experiencing more infections in subsequent waves.^8^ A pattern of infections spreading from urban to rural areas has also been observed in the USA^12^ and India.^13^ The greater reproductive opportunies afforded by denser populations in urban areas likely contributed to the initial spread of the virus.^14^ Proposed reasons for the subsequent impact on rural populations include a false sense of security due to initially low infection levels^15^ and increased resistance in such areas to preventative measures such as mask wearing.^16^

To update the seroprevalence data for Nigeria, we performed a seroprevalence survey at an urban and a rural site in Kaduna State during October and November 2021.

## Methods

Surveillance was carried out in accordance with the WHO’s Unity Studies Sero-Epidemiological Investigations Protocols.^17^ A sample size of 400 participants was chosen such that the maximum standard error of the estimate of seroprevalence will be 5% (when seroprevalence is 50%). We screened a total of 802 participants attending hospital outpatient units for reasons not related to COVID-19. Hospitals were chosen to maximize the limited resources for sample collection and provide a secure environment for staff and participants. However it is possible that such population groups are not representative of the general population, with potentially higher rates of underlying illness and increased likelihood of having received a SARS-CoV-2 vaccination. In situations where provision of a blood sample was part of participant’s hospital appointment, samples for this study were collected in a separate vacutainer from the same needle, thus minimizing venepuncture.

Samples were collected at Yusuf Dantsoho Memorial Hospital, Tudun-Wada, Kaduna City (a hospital with established collaborations with the authors), *n *=* *400, sampling dates 11 October 2021 to 5 November 2021. Kaduna City (population 1.6 million) is the fourth largest city in Nigeria. Yusuf Dantsoho Memorial Hospital is a secondary level health facility located in the centre of the city. It was selected for this study as it serves one of Kaduna State’s most densely populated urban areas.

Samples were also collected from Hajiya Gambo Suwaba General Hospital, Kofar-Gayan, Zaria (a hospital with established collaborations with the authors), *n *=* *402, sampling dates 12 October 2021 to 8 November 2021. Hajiya Gambo Suwaba General Hospital is a secondary level health facility serving a rural population. It was selected for this study as it enabled sample collection from a rural population without the security concerns that were present elsewhere in Kaduna State. During sample collection at this hospital, participants were asked to identify whether their attendance at the outpatient unit was due to: (i) donating blood; (ii) attending an antenatal clinic; or (iii) some other reason.

At the time of sample collection, Nigeria was approaching the end of a third wave of SARS-CoV-2 infection (with the peak of the third wave occurring in August 2021). Informed consent was confirmed via signatures or thumb prints, and parental consent was obtained for participants who were less than 18 years old. Participants with learning disabilities were excluded from the study.

Ethical approval for this study was obtained from Kaduna State Ministry of Health (MOH/ADM/744/VOL.1/1019 NHREC/17/03/2018). Participants completed a questionnaire that captured age, sex, number of people living in their household, SARS-CoV-2 vaccination history and 12-month history of symptoms previously associated with COVID-19 (aches and pains, diarrhoea, dry cough, fever, headache, loss of taste and/or smell, nausea and sore throat.^18^ Participants were also given the opportunity to indicate that they had not experienced any such symptoms in the previous 12 months.

Approximately 5 ml of venous blood were collected from each consenting participant. Tubes were left to clot at room temperature for 1 h, then centrifuged at 2500 rpm for 15 min. The resulting serum was analysed immediately. The CE-marked Biopanda COVID-19 IgM/IgG Rapid Test Kit [sensitivity >99% (95% CI: 93.3%–100%), specificity 98.6% (95% CI: 94.9%–99.8%)] was used to screen serum for antibodies, following the manufacturers’ instructions. The presence of IgM antibodies indicates a recent infection (7–28 days prior to sampling); IgG antibodies typically appear approximately 14 days after infection and endure for at least several months, providing some degree of immunity from further infection (Table 1).

**Table 1:** Interpretation of Biopanda COVID-19 IgM/IgG Rapid Test Kit results

Test result		Interpretation
IgM	IgG	
–	–	Seronegative
–	+	Seropositive, likely >28 days after infection
+	–	Seropositive, likely within 28 days of infection
+	+	Seropositive, likely 14–28 days after infection

Participants were grouped into four age categories, each representing approximately one-quarter of the Nigerian population, and based on the United Nations Department of Economic and Social Affairs data on the population of Nigeria by 5-year age groups:^19^ those who were less than 10 years of age (31.0% of the population); those aged 10–19 (23.1%); those aged 20–34 (22.4%); and those aged over 35 (23.5%).

### Data analysis

To estimate seroprevalence, we first determined unadjusted frequencies of positive tests as a proportion of the total sample size for the following groupings: location (Kaduna City or Kofar-Gayan); test type (IgG only, IgM only or antibody detection by either test); sex (female or male); age group (0–9, 10–19, 20–34 and 35+ years); SARS-CoV-2 vaccination status (have or have not received at least one dose of a SARS-CoV-2 vaccine); and symptoms reported (aches and pains, diarrhoea, dry cough, fever, headache, loss of taste and/or smell, nausea, sore throat, no symptoms). We used z-tests to compare seroprevalence of age groups, different locations and sexes. We used t tests to determine whether age and household size are associated with seropositivity. We used multivariate logistic regression (with age, sex, household size and SARS-CoV-2 vaccination status as covariates) to derive odds ratios and *P*-values for associations between reported symptoms and SARS-CoV-2 seropositivity. Variance inflation factors (VIF) were inspected for evidence of multi-collinearity in the analysis, and the Hosmer–Lemeshow test was used to check the fit of the model to the data. A VIF threshold of 5 was set for the exclusion correlated covariates: no covariates were excluded using this criterion. We used data on Nigerian population age distribution by sex from the United Nations Department of Economic and Social Affairs to obtain age- and sex-stratified estimates for overall seroprevalence. We compared these estimates with official SARS-CoV-2 infection data for Kaduna State.

## Results

In total, 802 participants (Table 2 and Supplementary Figure S1, available as Supplementary data at *IJE* online) were screened for the presence of SARS-CoV-2 antibodies in Kaduna City (urban site, *n *=* *400) and Kofar-Gayan (rural site, *n *=* *402) during October and November 2021. Table 3 and Supplementary Figure S2 (available as Supplementary data at *IJE* online) summarise antibody responses for Kaduna City and Kofar-Gayan.

**Table 2:** Participant demographics: Kaduna City and Kofar-Gayan

	Kaduna City	Kofar-Gayan
	Urban	Rural
Total	400	402
Female	233 (58.2%)	153 (38.1%)
Male	167 (41.8%)	248 (61.7%)
Sex not recorded	0 (0%)	1 (0.2%)
Age 0–9	55 (13.7%)	16 (4.0%)
Age 10–19	59 (14.7%)	43 (10.7%)
Age 20–34	108 (27.0%)	288 (71.6%)
Age 35+	145 (36.2%)	52 (12.9%)
Age not recorded	33 (8.2%)	3 (0.7%)
Mean age (years)	30.7	26.0
Median age (years)	26.0 (16.5–46.0)	24.0 (21.0–29.5)
Median household size	7.0 (5.0–9.0)	12.0 (8.0–17.0)
Received at least one dose of a SARS-CoV-2 vaccine	33 (8.2%)	16 (4.0%)
Antenatal clinic attenders	70	70 (17.4%)
Blood donors	140	140 (34.8%)
Attended hospital for other reason	192	192 (47.8%)
Reason for hospital attendance not recorded	400 (100.0%)	0 (0.0%)

For median age and median household size, figures in brackets represent the interquartile range.

**Table 3:** IgM and IgG seropositivity, Kaduna City and Kofar-Gayan

	Kaduna City (*n *=* *400)	Kofar-Gayan (*n *=* *402)	*P*-value
IgG positive only	129 (32.2%, 95% CI 27.6%–36.8%)	189 (47.0%, 95% CI 42.1%–51.9%)	<0.001
IgM positive only	23 (5.7%, 95% CI 3.4%–8.0%)	13 (3.2%, 95% CI 1.5%–4.9%)	0.085
IgG and IgM positive	18 (4.5%, 95% CI 2.5%–6.5%)	13 (3.2%, 95% CI 1.5%–4.9%)	0.352
All IgG positive	147 (36.7%, 95% CI 32.0%–41.4%)	202 (50.2%, 95% CI 45.3%–55.1%)	<0.001
All IgM positive	41 (10.2%, 95% CI 7.2%–13.2%)	26 (6.5%, 95% CI 4.1%–8.9%)	0.052
All positive	170 (42.5%, 95% CI 37.7%–47.3%)	215 (53.5%, 95% CI 48.6%–58.4%)	0.002
Negative	230 (57.5%, 95% CI 52.7%–62.3%)	187 (46.5%, 95% CI 41.6%–51.4%)	0.002

Z-tests were used to calculate *P*-values to compare subgroups. Confidence intervals were calculated as ±1.96 x standard error of the proportion who were seropositive.

### SAS-CoV-2 seroprevalence data for Kaduna city (urban site)

In Kaduna City, antibodies were detected in 170 participants (42.5%) of whom 41 (10.2% of all Kaduna City participants) were IgM-positive, suggestive of a recent infection. No detectable difference between seroprevalence rates in female and male participants was observed (*P *=* *0.064) (Table 4 and Supplementary Figure S3, available as Supplementary data at *IJE* online). Seroprevalence among participants who were aged 0–9 and 10–19 was lower (*P *<0.00001) than older age groups (Table 5 and Supplementary Figure S4, available as Supplementary data at *IJE* online), and the average age of seropositive participants (33.1 years) was greater (*P *=* *0.018) than those who were seronegative (28.7 years). No differences were observed between SARS-CoV-2 seropositive and seronegative participants in terms of household size. Vaccination against SARS-CoV-2 was reported in 33 participants (8.25%), 30 of whom reported receiving the AZD1222 (ChAdOx1 nCoV-19) vaccine (manufactured by AstraZeneca) and three reported having been vaccinated with Ad26.COV2.S (manufactured by Janssen). Seroprevalence among vaccinated participants (93.9%) was higher (*P *<0.00001) than among participants who had not received a vaccine (37.9%).

**Table 4:** Seropositivity in females and males Kaduna City and Kofar-Gayan

	Females	Males	*P*-value (identifying difference between sexes)
Kaduna City (urban)	90/233 (38.6%, 95% CI 32.3%–44.9%)	80/167 (47.9%, 95% CI 40.3%–55.5%)	0.064
Kofar-Gayan (rural)	80/153 (52.3%, 95% CI 44.4%–60.2%)	134/247 (54.3%, 95% CI 48.1%–60.5%)	0.704
*P*-value (identifying difference between locations)	0.008	0.204	

Z-tests were used to calculate *P*-values to compare subgroups. Confidence intervals were calculated as ±1.96 x standard error of the proportion who were seropositive.

**Table 5:** Seropositivity by age group, Kaduna City and Kofar-Gayan

Age group	Kaduna City		Kofar-Gayan	
	% positive	95% CI	% positive	95% CI
0–9	32.7	20.3%–45.1%	25.0	3.8%–46.2%
10–19	27.1	15.8%–38.4%	55.8	35.9%–75.7%
20–34	54.6	45.2%–64.0%	54.5	48.7%–60.3%
35+	49.7	41.6%–57.8%	51.9	33.0%–70.8%

Confidence intervals were calculated as ±1.96 × standard error of the proportion who were seropositive.

#### Symptoms (Kaduna City)

Participants reported experiencing an average of 1.6 of the eight common COVID-19 symptoms listed in the questionnaire. Dry cough, fever, loss of taste and/or smell, nausea and sore throat were associated with SARS-CoV-2 seropositivity (Table 6). SARS-Cov-2 seropositive participants reported more symptoms than those who were seronegative (*P *<0.001).

**Table 6:** SARS-CoV-2 antibody test results by symptoms reported, Kaduna City

Symptom (total number reporting)	SARS-CoV-2 seropositive (%)	Seropositive OR (95% CI)	*P*-value
Aches and pains (74)	57 (77.0%)	2.2 (0.9–5.9)	0.10
Diarrhoea (24)	9 (37.5%)	1.1 (0.4–3.4)	0.85
**Dry cough (67)**	**43 (64.2%)**	**3.1 (1.3–7.7)**	**0.01**
**Fever (112)**	**72 (64.3%)**	**2.1 (1.1–4.2)**	**0.03**
Headache (221)	95 (43.0%)	1.1 (0.5–2.2)	0.89
**Loss of taste and/or smell (72)**	**63 (87.5%)**	**24.0 (6.8–85.2)**	**8 × 10^−7^**
**Nausea (22)**	**15 (68.1%)**	**4.0 (1.2–13.7)**	**0.03**
**Sore throat (62)**	**53 (85.5%)**	**4.4 (1.5–13.0)**	**0.007**
No symptoms reported (68)	16 (23.5%)	1.2 (0.5–3.1)	0.69

Symptoms associated with seropositivity are highlighted in bold. Logistic regression (with age, sex, household size and SARS-CoV-2 vaccination status as covariates) was used to calculate odds ratios (ORs), 95% confidence intervals, and *P*-values (the odds ratios and *P*-values shown are adjusted for these covariates). The Hosmer–Lemeshow Test was used to test the fit of the model to the data: no evidence of a poor fit was found (*P *=* *0.81). Variance inflation factors (VIFs) were inspected for evidence of multicollinearity between variables and no evidence was found (maximum VIF = 2.1). ORs and *P*-values associated with with age, sex, household size and SARS-CoV-2 vaccination status are shown in Supplementary Table S1 (available as Supplementary data at *IJE* online). SARS-CoV-2 vaccination status was associated with positive antibody test result.

### SARS-CoV-2 seroprevalence data for Kofar-Gayan (rural site)

In Kofar-Gayan, antibodies were detected in 215 participants (53.5%), of whom 26 (6.5% of all Kofar-Gayan participants) were IgM seropositive (Table 3). No detectable differences in seroprevalence rates were detected when comparing female and male participants (Table 4 and Supplementary Figure S3), age groups (Table 5 and Supplementary Figure S4), reasons for attending the hospital outpatient unit (Table 7 and Supplementary Figure S5, available as Supplementary data at *IJE* online) or household sizes. Vaccination against SARS-CoV-2 was reported in 16 participants (4.0%), all of whom reported receiving the AZD1222 (ChAdOx1 nCoV-19) vaccine. Seroprevalence among vaccinated and unvaccinated participants was 75.0% and 52.6%, respectively: a difference that was not statistically significant (*P *=* *0.08).

**Table 7:** Seropositivity by reason for attending the hospital outpatient unit, Kofar-Gayan

Reason for attending outpatient unit	*n*	% positive	95% CI
Antenatal clinic	70	52.9%	41.2%–64.6%
Blood donation	140	53.6%	45.3%–61.9%
Other reason	192	53.6%	46.5%–60.7%

Confidence intervals were calculated as ±1.96 x standard error of the proportion who are seropositive.

#### Symptoms (Kofar-Gayan)

Participants reported experiencing an average of 3.7 of the eight common COVID-19 symptoms listed in the questionnaire. Headache and loss of taste and/or smell were associated with seropositivity (Table 8). Seropositive participants reported experiencing more symptoms than those who were seronegative (*P *=* *0.010).

**Table 8:** SARS-CoV-2 antibody test results by symptoms reported, Kofar-Gayan

Symptom (total number reporting)	SARS-CoV-2 seropositive (%)	Seropositive OR (95% CI)	*P*-value
Aches and pains (365)	199 (54.5%)	1.0 (0.4–2.8)	0.92
Diarrhoea (33)	16 (48.5%)	0.6 (0.3–1.4)	0.28
Dry cough (157)	86 (54.8%)	1.1 (0.6–1.9)	0.80
Fever (333)	172 (51.7%)	0.5 (0.2–1.0)	0.04
**Headache (373)**	**204 (54.7%)**	**6.1 (1.5–25.1)**	**0.01**
**Loss of taste and/or smell (62)**	**50 (80.6%)**	**4.5 (2.2–9.5)**	**6 × 10^−5^**
Nausea (137)	74 (54.0%)	1.3 (0.7–2.5)	0.41
Sore throat (46)	31 (67.4%)	1.7 (0.8–3.4)	0.17
No symptoms reported (14)	6 (42.9%)	3.4 (0.4–25.9)	0.24

Symptoms associated with seropositivity are highlighted in bold. Logistic regression (with age, sex, household size and SARS-CoV-2 vaccination status as covariates) was used to calculate odds ratios (ORs), 95% confidence intervals and *P*-values (the odds ratios and *P*-values shown are adjusted for these covariates). The Hosmer–Lemeshow Test was used to test for evidence of a poor fit of the model to the data: no evidence of a poor fit was found (*P *=* *0.73). Variance inflation factors (VIFs) were inspected for evidence of multicollinearity between variables and no evidence was found (maximum VIF = 2.6). ORs and *P*-values associated with age, sex, household size and SARS-CoV-2 vaccination status are shown in Supplementary Table S2 (available as Supplementary data at *IJE* online).

#### Comparison of SARS-CoV-2 seroprevalence data for Kaduna City and Kofar-Gayan

Unadjusted seroprevalence was higher in Kofar-Gayan than Kaduna City (*P *=* *0.002). No difference in seroprevalence rates was detected in male participants at the two sites; however seroprevalence rates in females in Kaduna City were lower than in Kofar-Gayan (*P *=* *0.008) (Table 4). Participants in Kaduna were more likely to have been vaccinated against SARS-CoV-2 (*P *=* *0.011). Participants in Kofar-Gayan reported experiencing more symptoms associated with COVID-19 (*P *<0.001).

## Discussion

Serological data for SARS-CoV-2 infection in sub-Saharan Africa are limited. As of November 2021, one previous peer-reviewed serological survey has taken place in Nigeria,^11^ and the country has subsequently experienced two further waves of SARS-CoV-2 infection.^2^ Although the surveillance described here was limited to two sites, it provides evidence on the proportion of the Nigerian population who have been exposed to the virus. The surveillance method deployed does not, however, differentiate between seropositivity as a result of infection or as a result of vaccination. Other caveats include those individuals who are unable to mount a detectable antibody response, and the fact that SARS-CoV-2 antibody titres have been shown to decline in the months following exposure. Recently, evidence has emerged suggesting that cross-reactivity with other circulating viruses or parasites in the African subcontinent might impact on SARS-CoV-2 serological surveys.^20^^,^^21^ The extent of cross-reactivity is unclear; however it should be noted that the SARS-CoV-2 serological surveys carried out early in the pandemic typically reported low seroprevalence. The average seroprevalence from the six SARS-CoV-2 serosurveys carried out in sub-Saharan Africa between March 2020 and June 2020 was less than 3%,^3^ suggesting that the impact of cross-reactivity on these surveys was limited.

Samples obtained from hospital outpatient units cannot be considered a random sample from the general population, as they are likely to have an overrepresentation among patients in groups with potentially higher rates of underlying illness and increased likelihood of having been vaccinated against SARS-CoV-2. However, it is not likely that patients attending the unit for antenatal clinics or to donate blood have overrepresentation in such groups. In Kofar-Gayan, no differences in seroprevalence were detected between patients attending the outpatient unit for antenatal clinics, to donate blood or for other reasons. Comparable data from Kaduna City were not available.

Due to the delay between exposure and development of SARS-CoV-2 antibodies, it is likely the seropositive participants of this survey will have been infected by or vaccinated against the virus by 27 September 2021 (2 weeks prior to the commencement of sampling).

Across the two sites examined in this study, the age- and sex-adjusted seroprevalence was 43.7%, and the age- and sex-adjusted seroprevalence among unvaccinated participants was 42.2%. The participants of this survey might not be representative of the general population, and the extent to which SARS-CoV-2 seroprevalence in the region varies is unclear. However if the seroprevalence data reported here are representative of Kaduna State (population 8.25 million^22^) it would indicate at least 3.48 million SARS-CoV-2 infections since the pandemic began: a figure 359 times greater than the state’s 9695 confirmed cases as of 27 September 2021.^22^

During sample and data collection, the proportion of the Nigerian population who had received at least one dose of a SARS-CoV-2 vaccine was between 2.3% and 2.7%.^2^ Self-reporting of vaccination in this survey indicated that 8.25% of participants in Kaduna City and 4.0% of participants in Kofar-Gayan had received at least one dose of a vaccine. This indicates that participants were not representative of the overall Nigerian population in terms of vaccination status. The factors responsible are not clear, although in Nigeria older age groups were prioritized for SARS-CoV-2 vaccination^23^ and the median age of participants in this survey (25) was older than the reported median age for the Nigerian population (18).^24^ The observed differences in vaccination rates between Kaduna City and Kofar-Gayan suggest potentially uneven distribution of vaccines between urban and rural populations in Nigeria. Unsurprisingly, in Kaduna City seroprevalence rates were higher among vaccinated participants than among those who had not been vaccinated. A statistically significant difference was not observed in Kofar-Gayan; however this was likely due to the limitations of statistical power in a site in which 16/400 participants were vaccinated.

In contrast to previous seroprevalence data comparing urban and rural areas,^8^ SARS-CoV-2 antibody seroprevalence was higher in Kofar-Gayan than in Kaduna City. Variation between geographical locations in one country is a common feature of the serological surveillance of respiratory viruses,^25^ with multiple factors promoting these differences. One possible factor is household size. Larger household sizes have previously been implicated in the spread of the virus.^26^ Participants in Kofar-Gayan had a median household size of 12 compared with those in Kaduna City (7), suggesting a possible explanation for this variation. Within Kaduna City, the lower seroprevalence observed in females and younger age groups highlights population subgroups who, without protective antibodies, may be susceptible to future waves of SARS-CoV-2 infection and who might benefit from targeted public health strategies.

Symptoms identified as being associated with COVID-19 reported here are consistent with previous studies. Participants in Kofar-Gayan reported more symptoms than those in Kaduna City, and in Kofar-Gayan, fever was associated with being seronegative. These observations could be due to increased circulation of other pathogens or other socioeconomic factors.

On 26 November 2021, WHO classified SARS-CoV-2 variant B.1.1.529 a variant of concern, and designated it with the name Omicron.^27^ Preliminary data indicate that Omicron is likely to replace Delta as the world’s predominant SARS-CoV-2 variant,^28^ a change that would likely result in increased rates of SARS-CoV-2 infection. Obtaining baseline measurements that record the proportion of the population with some antibody response to previous variants of SARS-CoV-2 immediately prior to the introduction of Omicron will inform intervention strategies.

### Strengths of this study

Despite being Africa’s most populated country, SARS-CoV-2 seroprevalence data for Nigeria are extremely limited: the only previous peer-reviewed data were obtained from samples collected in December 2020. This study provides an updated estimate for SARS-CoV-2 seroprevalence in Nigeria and enabled seroprevalence immediately prior to the spread of the Omicron variant to be estimated. The sampling strategy and inclusion of a questionnaire enabled seroprevalence rates to be compared in terms of location (urban or rural), age group, sex, household size and SARS-CoV-2 vaccination status, and thus the identification of groups within the population who have lower seroprevalence and might therefore be more susceptible to subsequent waves of SARS-CoV-2 infection. Additionally, this study presents associations between SARS-CoV-2 seropositivity and symptoms in a Nigerian environmental background. By comparing these data with figures for confirmed SARS-CoV-2 cases, we were able to quantify the underestimation of the burden of the virus on Kaduna State.

### Limitations of this study

As sampling was carried out in hospitals, the extent to which the seroprevalence measured is representative of the general population is unclear, particularly in Kaduna City where the proportion of samples from blood donors and antenatal clinic attendees was not known. The study contains additional limitations that should be addressed in subsequent work. Recording data on additional sociodemographic factors (such as education level, occupation, and indicators of deprivation) and health factors (such as underlying health conditions and additional information on the participant’s reason for attending hospital) would have provided further information on factors driving seroprevalence. Collection of data at additional sites would have confirmed whether seroprevalence observed was typical for Kaduna State and for Nigeria. Collection of additional samples would provide improved statistical power and enabled seroprevalence differences between ages to be examined in more detail. Collection of samples at additional time points would enable temporal seroprevalence changes to be tracked.

## Conclusions

This study provides SARS-CoV-2 seroprevalence data for the most populated sub-Saharan African country. It provides detailed information related to seroprevalence for two locations in Kaduna State. Further studies are required to ascertain whether the seroprevalence reported is consistent across the country and to record longitudinal seroprevalence changes. Our work indicates that infection rates in Kaduna State, Nigeria, might be at least 359 times greater than figures for confirmed cases and provides evidence that as of October/November 2021, at least 43.7% of the population have been exposed to the virus via infection or vaccination and that approximately 56% did not carry SARS-CoV-2 antibodies. The work presented here will inform public health policy and deployment strategies for testing, treatment and vaccination in Nigeria and will provide a record of SARS-CoV-2 seroprevalence in Nigeria during October/November 2021. Additionally, it will provide a baseline for SARS-CoV-2 seroprevalence in Nigeria immediately prior to the spread of the Omicron variant.

## Ethics approval

This work was approved by the ethics committee of Kaduna State Ministry of Health (MOH/ADM/744/VOL.1/1019 NHREC/17/03/2018).

## Data availability

The data underlying this article cannot be shared publicly for the privacy of individuals that participated in the study.

## Supplementary data

Supplementary data are available at *IJE* online.

## Author contributions

G.C. conducted surveillance, curated the data, performed data analysis and reviewed and edited the manuscript. J.K. managed and coordinated activity planning, execution and project administration. J.Y. conducted surveillance and performed data analysis. HN. provided methodology and supervision for statistical analyses and reviewed and edited the manuscript. A.M. reviewed and edited the manuscript. W.A. designed and developed the study and the methodology underpinning it, acquired funding, provided supervision and wrote the manuscript.

## Funding

This work was funded by the University of Glasgow COVID-19 Researcher Support Scheme.

## Conflict of interest

None declared.

## Supplementary Material

dyac141_Supplementary_Data

## References

[1] Cucinotta D , Vanelli M. WHO declares COVID-19 a pandemic. Acta Biomed 2020;91:157–60. 32191675 10.23750/abm.v91i1.9397 PMC7569573

[2] Ritchie H , Mathieu E, Rodes-Guirao L et al *Coronavirus Pandemic (COVID-19)*. 2020. http://ourworldindata.org/coronavirus (10 May 2022, date last accessed).

[3] Arora RK , Joseph A, Van Wyk J et al SeroTracker: a global SARS-CoV-2 seroprevalence dashboard. Lancet Infect Dis 2021;21:E75–76. 32763195 10.1016/S1473-3099(20)30631-9 PMC7402646

[4] Gudina EK , Ali S, Girma E et al Seroepidemiology and model-based prediction of SARS-CoV-2 in Ethiopia: longitudinal cohort study among front-line hospital workers and communities. Lancet Glob Health 2021;9:E1517–27. 34678196 10.1016/S2214-109X(21)00386-7 PMC8525918

[5] Nyagwange J , Kutima B, Mwai K et al Comparative performance of WANTAI ELISA for total immunoglobulin to receptor binding protein and an ELISA IgG to spike protein in detecting SARS-CoV-2 antibodies in Kenyan populations. J Clin Virol 2022;146:105061. 34973474 10.1016/j.jcv.2021.105061 PMC8711170

[6] Mandolo J , Msefula J, Henrion MYR et al SARS-CoV-2 exposure in Malawian blood donors: an analysis of seroprevalence and variant dynamics between January 2020 and July 2021. BMC Med 2021;19:303. 34794434 10.1186/s12916-021-02187-y PMC8601780

[7] Fryatt A , Simms V, Bandason T et al Community SARS-CoV-2 seroprevalence before and after the second wave of SARS-CoV-2 infection in Harare, Zimbabwe. EClinicalMedicine 2021;41:101172. 34723165 10.1016/j.eclinm.2021.101172 PMC8542175

[8] Kleynhans J , Tempia S, Wolter N et al SARS-CoV-2 seroprevalence in a rural and urban household cohort during first and second waves of infections, South Africa, July 2020-March 2021. Emerg Infect Dis 2021;41:101172. 10.3201/eid2712.211465 PMC8632160 34477548

[9] Somboro AM , Cissoko Y, Camara I et al High SARS-CoV-2 seroprevalence among healthcare workers in Bamako, Mali. Viruses 2022;14:102. 35062306 10.3390/v14010102 PMC8780908

[10] Usuf E , Roca A. Seroprevalence surveys in sub-Saharan Africa: what do they tell us? Lancet Glob Health 2021;9:E724–25. 33711261 10.1016/S2214-109X(21)00092-9

[11] Okpala OV , Dim CC, Ugwu CI et al Population seroprevalence of SARS-CoV-2 antibodies in Anambra State, South-East, Nigeria. Int J Infect Dis 2021;110:171–78. 34293489 10.1016/j.ijid.2021.07.040 PMC8288214

[12] Paul R , Arif AA, Adeyemi O, Ghosh S, Han D. Progression of COVID-19 from urban to rural areas in the United States: a spatiotemporal analysis of prevalence rates. J Rural Health 2020;36:591–601. 32602983 10.1111/jrh.12486 PMC7361905

[13] George CE , Inbaraj LR, Rajukutty S et al Seroprevalence of COVID-19 infection among vaccine naïve population after the second surge (June 2020) in a rural district of South India: a community-based cross-sectional study. PloS One 2022;17:e0265236. 35271669 10.1371/journal.pone.0265236 PMC8912234

[14] Delamater PL , Street EJ, Leslie TF, Yang T, Jacobsen KH. Complexity of the basic reproduction number (R0). Emerg Infect Dis 2019;25:1–4. 10.3201/eid2501.171901 PMC6302597 30560777

[15] Peters DJ. Community susceptibility and resiliency to COVID-19 across the rural-urban continuum in the United States. J Rural Health 2020;36:446–56. 32543751 10.1111/jrh.12477 PMC7323251

[16] Haischer MH , Beilfuss R, Hart MR et al Who is wearing a mask> gender-, age-, and location-related difference during the COVID-19 pandemic. PloS One 2020;15:e0240785. 33057375 10.1371/journal.pone.0240785 PMC7561164

[17] World Health Organization. Population-based Age-stratified Seroepidemiological Investigation Protocol for Coronavirus 2019 (Covid-19) Infection, Version 2.0. 2020. https://www.who.int/publications/i/item/WHO-2019-nCoV-Seroepidemiology-2020.2 (10 May 2022, date last accessed).

[18] Centers for Disease Control and Prevention. *COVID-19*. 2020. https://www.cdc.gov/coronavirus/2019-ncov/ (10 May 2022, date last accessed).

[19] United Nations Department of Economic and Social Affairs. Population Dynamics *.* https://population.un.org/wpp/Download/Standard/Population/ (10 May 2022, date last accessed).

[20] Ndaye AN , Hoxha A, Madinga J et al Challenges in interpreting SARS-CoV-2 serological results in African countries. Lancet Glob Health 2021;9:E588–89. 33609481 10.1016/S2214-109X(21)00060-7 PMC7906714

[21] Ashworth J , Mathie D, Scott F et al Pre-SARS-CoV-2 human sera reacts with peptides from all the 7 human coronaviruses: peptide microarray IgM and IgG screening. Lancet 2022; doi:10.2139/ssrn.4010886.

[22] Nigeria Centre for Disease Control. COVID-19 Situation Report. Weekly epidemiological report 51, Epi week 39, 27 September–3 October 2021. https://ncdc.gov.ng/diseases/sitreps/?cat=14&name=An%20update%20of%20COVID-19%20outbreak%20in%20Nigeria (10 May 2022, date last accessed).

[23] World Health Organization Regional Office for Africa. Nigerian Health Workers Take Country’s First COVID-19 Vaccine. 2021. https://www.afro.who.int/news/nigerian-health-workers-take-countrys-first-covid-19-vaccine (10 May 2022, date last accessed).

[24] Ritchie H , Roser M. *Age Structure.* http://ourworldindata.org/age-structure. (10 May 2022, date last accessed).

[25] Adamson WE , Maddi S, Robertson C et al Pandemic influenza A(H1N1) virus in Scotland: geographically variable immunity in Spring 2010, following the winter outbreak. Euro Surveill 2010 2009;15:19590. 20576237

[26] Hallal PC , Hartwig FP, Horta BL et al SARS-CoV-2 antibody seroprevalence in Brazil: results from two successive nationwide serological household surveys. Lancet Glob Health 2020;8:E1390–98. 32979314 10.1016/S2214-109X(20)30387-9 PMC7511212

[27] World Health Organization. Classification *of Omicron (B.1.1.529): SARS-CoV-2 Variant of Concern.* 2021. https://www.who.int/news/item/26-11-2021-classification-of-omicron-(b.1.1.529)-sars-cov-2-variant-of-concern (10 May 2022, date last accessed).

[28] Torjesen I. Covid restrictions tighten as omicron cases double every two to three days. BMJ 2021;375:n3051. 34887256 10.1136/bmj.n3051

