# Supplementary material for: SARS-CoV-2 seroprevalence at urban and rural sites in Kaduna State, Nigeria, during October/November 2021, immediately prior to detection of the Omicron variant
Source: Int J Epidemiol. 2022 Jun 30;51(5):1361–70. doi: 10.1093/ije/dyac141 (PMC9278217; doi:10.1093/ije/dyac141)
Supplement: dyac141_Supplementary_Data [file dyac141_supplementary_data.docx]

**Supplementary data**

**Figure S1:** Participants recruited at each site, and antibody responses.


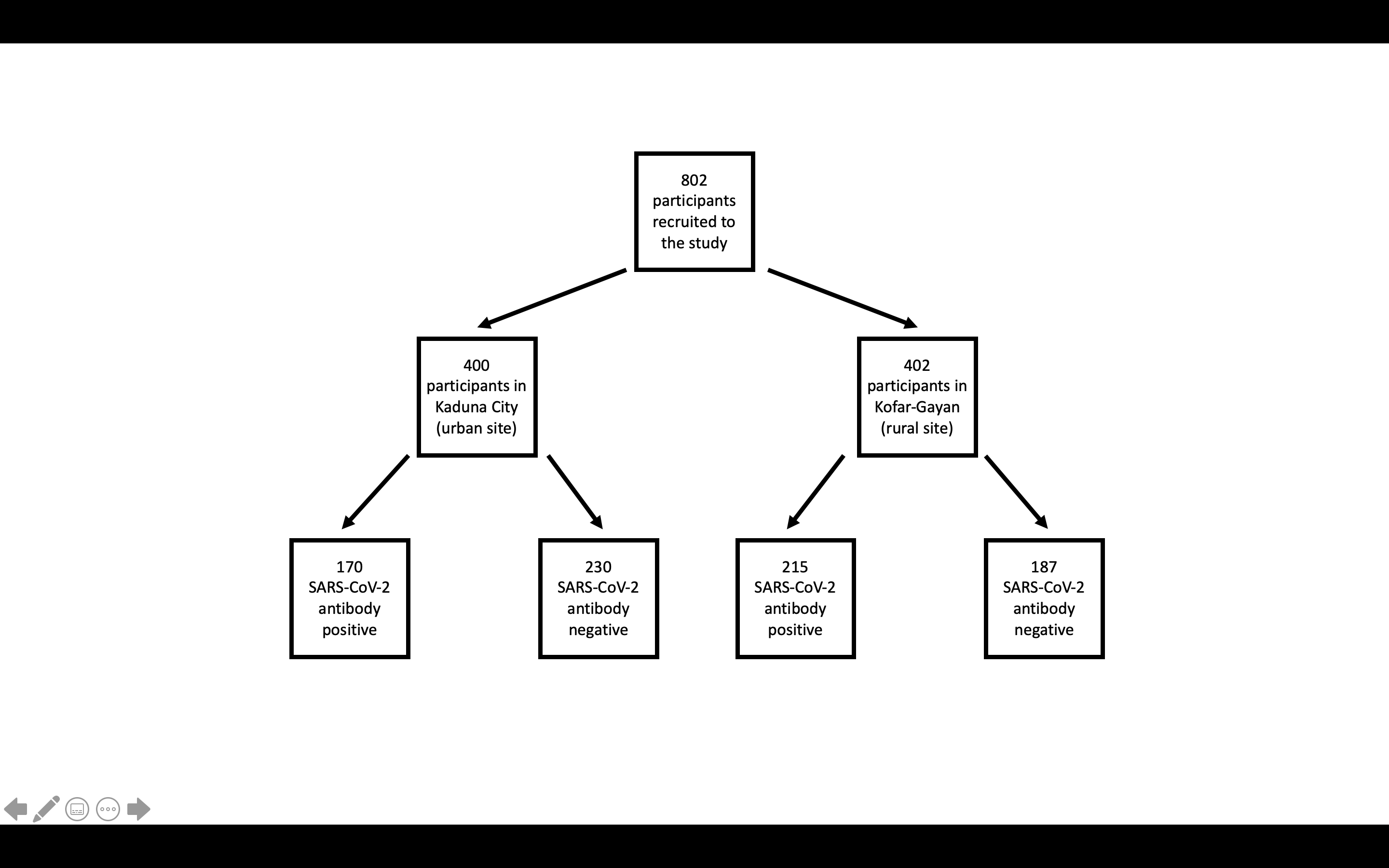


**Figure S2:** IgM and IgG seropositivity, Kaduna City and Kofar-Gayan.

Confidence intervals were calculated as ±1.96 x standard error of the proportion who were seropositive.

**Figure S3:** Seropositivity in females and males Kaduna City and Kofar-Gayan.

Confidence intervals were calculated as ±1.96 x standard error of the proportion who were seropositive.

**Figure S4:** Seropositivity by age group, Kaduna City and Kofar-Gayan.

Confidence intervals were calculated as ±1.96 x standard error of the proportion who were seropositive.

**Figure S5:** Seropositivity by reason for attending the hospital outpatient unit, Kofar-Gayan.

Confidence intervals were calculated as ±1.96 x standard error of the proportion who are seropositive.

**Table S1**: Associations of SARS-CoV-2 antibody test results with variables other than symptoms (i.e. age, sex, household size, and SARS-CoV-2 vaccination status in Kaduna City.

| Covariate | Seropositive OR (95% CI) | *P*-value |
| --- | --- | --- |
| Age | 0.99 (0.98 – 1.01) | 0.99 |
| (Male) sex | 1.61 (0.94 – 2.76) | 0.08 |
| Household size | 0.97 (0.91 – 1.10) | 0.97 |
| Received SARS-CoV-2 vaccine | 14.57 (3.11 – 68.30) | 0.0007 |

**Table S2**: Associations of SARS-CoV-2 antibody test results with variables other than symptoms (i.e. age, sex, household size, and SARS-CoV-2 vaccination status in Kofar-Gayan.

| Covariate | Seropositive OR (95% CI) | *P*-value |
| --- | --- | --- |
| Age | 1.01 (0.99 – 1.03) | 0.36 |
| (Male) sex | 0.96 (0.56 – 1.64) | 0.88 |
| Household size | 1.01 (0.98 – 1.04) | 0.58 |
| Received SARS-CoV-2 vaccine | 3.22 (0.96 – 10.82) | 0.06 |
